# Differential geographic patterns in song components of male Albert’s lyrebirds

**DOI:** 10.1002/ece3.7225

**Published:** 2021-02-14

**Authors:** Fiona Backhouse, Anastasia H. Dalziell, Robert D. Magrath, Aaron N. Rice, Taylor L. Crisologo, Justin A. Welbergen

**Affiliations:** ^1^ The Hawkesbury Institute for the Environment Western Sydney University Richmond NSW Australia; ^2^ Centre for Sustainable Ecosystem Solutions School of Earth, Atmospheric and Life Sciences University of Wollongong Wollongong NSW Australia; ^3^ Fuller Evolutionary Biology Program Cornell Lab of Ornithology Cornell University Ithaca NY USA; ^4^ Macaulay Library Cornell Lab of Ornithology Cornell University Ithaca NY USA; ^5^ Center for Conservation Bioacoustics Cornell Lab of Ornithology Cornell University Ithaca NY USA; ^6^ Research School of Biology the Australian National University Canberra ACT Australia

**Keywords:** bird song, geographic variation, lyrebird, *Menuraalberti*, song function, song structure

## Abstract

Geographic variation in bird song has received much attention in evolutionary studies, yet few consider components within songs that may be subject to different constraints and follow different evolutionary trajectories. Here, we quantify patterns of geographic variation in the socially transmitted “whistle” song of Albert's lyrebirds (*Menura alberti*), an oscine passerine renowned for its remarkable vocal abilities. Albert's lyrebirds are confined to narrow stretches of suitable habitat in Australia, allowing us to map likely paths of cultural transmission using a species distribution model and least cost paths. We use quantitative methods to divide the songs into three components present in all study populations: the introductory elements, the song body, and the final element. We compare geographic separation between populations with variation in these components as well as the full song. All populations were distinguishable by song, and songs varied according to the geographic distance between populations. However, within songs, only the introductory elements and song body could be used to distinguish among populations. The song body and final element changed with distance, but the introductory elements varied independently of geographic separation. These differing geographic patterns of within‐song variation are unexpected, given that the whistle song components are always produced in the same sequence and may be perceived as a temporally discrete unit. Knowledge of such spatial patterns of within‐song variation enables further work to determine possible selective pressures and constraints acting on each song component and provides spatially explicit targets for preserving cultural diversity. As such, our study highlights the importance for science and conservation of investigating spatial patterns within seemingly discrete behavioral traits at multiple levels of organization.

## Graphical abstract

Male Albert's lyrebirds show geographic variation in their species‐specific whistle song, with acoustic differences between populations increasing with geographic distance. However, three discrete components within the song, the introduction, the body, and the final element, show contrasting patterns of spatial variation. This suggests that the same evolutionary drivers are not acting on each song component, demonstrating the importance of investigating spatial patterns in bird song at multiple levels of organization.

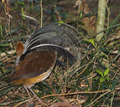

## 1 INTRODUCTION

Geographic variation in bird song is often used as a framework to examine patterns and processes of evolution in nature (Podos & Warren, 2007). This is in part because, as a learnt trait, it has the potential to evolve faster than purely genetic traits, and so exhibits considerable spatiotemporal variation both within and between species (Lynch, 1996; Mason et al., 2017), and may even lead to eventual speciation (Danner et al., 2011, 2017). Bird song can vary geographically for several reasons, including environmental (Nicholls & Goldizen, 2006), genetic or morphological (Badyaev et al., 2008; Derryberry, 2009), or cultural influences (Danner et al., 2011; Lynch, 1996). When variation is driven purely by stochastic processes such as genetic or cultural drift, we may expect differences in song to increase broadly with geographic distance between locations (Campbell et al., 2010; Irwin et al., 2008; Podos & Warren, 2007). On the other hand, local selection pressures and constraints, or a very high within‐population diversity, may act independently or in conjunction with random drift and lead to more complex patterns of acoustic variation (Cardoso & Reichard, 2016; Irwin, 2000; Podos & Warren, 2007).

While geographic variation in song has been studied in a broad range of bird species, independent variation of components within the song and the information this may provide about song evolution has been the subject of few studies (Benedict & Bowie, 2012; Lee et al., 2019; Nelson, 2017; Roach & Phillmore, 2017; Williams et al., 2013). Songs presented as a discrete unit may in fact comprise a set of signaling components, each with different mechanistic and functional constraints, and therefore subject to different selection pressures (Gil & Gahr, 2002; Richards, 1981). Some components may be physiologically difficult to produce, thereby limiting the extent of potential variation (Gil & Gahr, 2002). More complex components may be difficult to imitate accurately, leading to greater variation (Nelson, 2017). Different components may be important for inter‐ versus intraspecific communication (Dalziell & Cockburn, 2008; Leitão & Riebel, 2003) or may encode different kinds of information (Nelson & Poesel, 2007; Richards, 1981). Investigating patterns of variation within songs will provide clues on the evolution and functional significance of different song components (Lee et al., 2019).

Species with small population sizes and fragmented ranges are of particular interest in questions of geographic variation as they are more prone to both genetic and cultural isolation, and hence reproductive isolation; therefore, it is imperative to understand mechanisms of variation in these systems (Fayet et al., 2014; Koetz et al., 2007; Laiolo, 2010). Furthermore, habitat fragmentation may impede genetic flow between populations as well as cultural transmission of songs, leading to reduced song sharing between isolated individuals or populations (Keyghobadi, 2007; Laiolo & Tella, 2005). Recently, conservation efforts have moved from a focus purely on genetic and morphological diversity to consider behavioral diversity as well. One method is to identify “culturally significant units” which may aid in conservation planning by identifying culturally important or unique populations on which to focus efforts (Ryan, 2006). This is important both in terms of understanding cultural and therefore potential genetic exchange between populations, and in preserving unique cultures for their own sake (Caro & Sherman, 2012; Ryan, 2006). Despite this, few studies on song variation have been conducted on species considered to be at risk, such as those with small populations sizes and small or fragmented ranges (But see Koetz et al., 2007; Parker et al., 2012; Pavlova et al., 2012; Sebastián‐González & Hart, 2017).

The Albert's lyrebird (*Menura alberti*) is an ideal species for investigating geographic variation in song. Albert's lyrebirds are a sedentary species with limited dispersal abilities, making movement over large areas unlikely. Albert's lyrebirds have an extremely restricted range largely composed of narrow stretches of rainforest in eastern Australia that have experienced a high degree of habitat loss and fragmentation (BirdLife International, 2016), which may have promoted acoustic differences between populations. The restriction of suitable habitat within the Albert's lyrebird's range also allows us to easily map out likely paths of genetic or cultural transmission. Furthermore, Albert's lyrebirds are one of only two extant species of the Menuridae, an oscine passerine family known for their diverse vocal repertoires including highly accurate vocal mimicry in both species (Dalziell & Magrath, 2012; Dalziell & Welbergen, 2016; Putland et al., 2006; Robinson & Curtis, 1996; Zann & Dunstan, 2008). The species’ extraordinary mimetic ability means that morphological and cognitive constraints are likely to have little impact on vocal abilities and are therefore unlikely to drive variation among individuals or populations. Previous research on the Albert's lyrebird has found evidence of local “dialects” of species‐specific song (Robinson & Curtis, 1996), yet to date there has been little formal research on the Albert's lyrebird, and much of the work done has involved only qualitative descriptions of songs (e.g., Robinson & Curtis, 1996).

In order to better understand processes of cultural variation in the Albert's lyrebird, we investigated the geographic variation in a distinctive, socially transmitted, species‐specific song we call the “whistle song” (after Dalziell & Welbergen, 2016; Zann & Dunstan, 2008). To examine how the whistle song varies among local populations, we systematically sampled across the species range and examined the extent to which populations had culturally distinct whistle songs. We further examined geographic patterns of acoustic variation at two scales: at the level of the full whistle song, and at the level of structurally discrete components found within the whistle song. If variation in the whistle song is caused by cultural or genetic drift, then acoustic differences between populations are expected to increase with geographic distance. On the other hand, if whistle songs are subject to local selection pressures or constraints, then differences between populations may not be correlated with geographic distance. If there are different patterns of spatial variation within each song component, then the song components cannot be under the same selection pressures or constraints and are therefore following independent evolutionary trajectories.

## 2 MATERIAL AND METHODS

### 2.1 Study species

Albert's lyrebirds are large (930 g) oscine passerines that have a small species range with varying degrees of fragmentation (Higgins et al., 2001; Robinson & Curtis, 1996). Individual males are territorial and are largely solitary except during sexual interactions or territorial encounters (Higgins et al., 2001). Males can be distinguished from females and juveniles by their longer, more extravagant tail including highly filamented feathers (Higgins et al., 2001). Male Albert's lyrebirds perform elaborate multicomponent displays during the months of March to August, when they perform dance‐like displays on “display platforms” within their territory in conjunction with their own song and sequences of vocal mimicry of other species ("sequential mimicry": Curtis, 1972; Higgins et al., 2001).

### 2.2 Study sites

We studied Albert's lyrebirds throughout the species range in subtropical and temperate rainforest and wet sclerophyll forest in northeast New South Wales and southeast Queensland, Australia. Albert's lyrebird habitat is largely restricted to the mountain ranges of the ancient Tweed volcano and surrounding volcanic ridges, found between 28.89° and 27.89°S and 152.36° and 153.46°E. The area is characterized by cool, dry winters and warm, wet summers with an annual rainfall of approximately 1,560 mm (Bureau of Meteorology, 2019).

Data for this study were collected from six different sites that vary in their degree of isolation: Koonyum Range in Mt Jerusalem National Park (28.53°S, 153.40°E), Border Ranges National Park (28.38°S, 153.08°E), Lamington National Park (Binna Burra section, 28.21°S, 153.19°E), Tamborine National Park (27.93°S, 153.19°E), a small patch of forest near Killarney, QLD (28.31°S, 152.40°E), and the Goomburra section of Main Range National Park (27.97°S, 152.39°E). These sites encompass the variety of habitat types found in the Albert's lyrebird's range and include populations from the two northern extremes of their range, one population toward the southeast of their range, and three intermediate populations.

### 2.3 Field methods

We took field recordings of six male Albert's lyrebirds near Killarney during the breeding season (May – July) of 2016, five each from the Border Ranges, Mt Jerusalem, Lamington, and Tamborine during the breeding season of 2018, and six from Goomburra during the breeding season of 2018. Recordings were made by following individuals as closely as possible without disturbing them, usually resulting in a distance of 15–30 m from the focal bird. Individuals could be identified by location, as they often sang from the same display platform each morning; camera footage of two individuals identifiable by a missing median feather were consistently filmed on the same platforms. While only males are known to sing extended bouts of mimicry and whistle song (Higgins et al., 2001), the sex of the focal birds was confirmed visually or from footage from camera traps recorded at display platforms. All recordings were taken on or near their display platforms as the birds were also performing sequences of mimicry, an important part of their sexual display (Higgins et al., 2001). Recordings were made using a handheld Sennheiser ME 66/K6 shotgun microphone and a Marantz PMD 661 with a 94 kHz sample rate and 24‐bit depth.

### 2.4 Study songs

**Figure 1:**
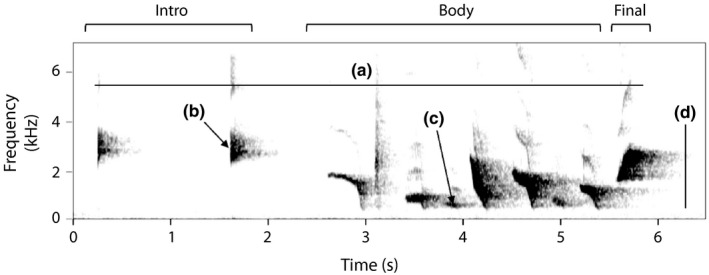
A spectrogram of a representative whistle song from the Border Ranges population with the song components marked out and four song‐level measurements calculated from the element‐level measurements: (a) Song duration, (b) Max frequency, (c) Min frequency, (d) Song bandwidth. Produced with a "Hann" display and FFT 1,722

Albert's lyrebirds have a broad range of vocalizations including mimicry, but we focus on a striking, species‐specific song we here refer to as the “whistle song” (Figure 1; also known as “territorial song”: Robinson & Curtis, 1996). The whistle song is a loud song of approximately five seconds in duration, of unconfirmed behavioral function. Male Albert's lyrebirds usually begin the dawn chorus by singing whistle songs before incorporating sequences of vocal mimicry (Higgins et al., 2001). The whistle song is then repeated at irregular intervals throughout the mimicry, usually with a short (3–10 s) silence before and after the whistle song (Backhouse, Pers. Obs.). The mimicry during these singing bouts is thought to be directed toward females (Higgins et al., 2001), whereas the whistle song may be directed toward other males, as males have been heard responding with whistle song to the whistle songs of neighboring males (Robinson & Curtis, 1996, Backhouse, Pers. Obs.). Whistle songs usually begin with 1–3 “introductory elements” that in some populations are considered mimicry of other species, often Australian king parrots (*Alisterus scapularis*) or grey goshawks (*Accipiter novaehollandiae*) (Robinson & Curtis, 1996), which are followed after a short pause (1.28 ± 0.85 s) by longer, lyrebird‐specific elements (Robinson & Curtis, 1996). Our preliminary analysis indicated that in all populations, after the introductory elements, there was both a highly variable middle (“body”) section followed by a distinctive final element (Figure 1).

### 2.5 Acoustic measurements

Acoustic measurements were obtained for individual song elements, the smallest unit within the song, which could be identified as continuous lines on the spectrogram (Catchpole & Slater, 2008; Figure 1). Elements were measured in Raven Pro 64 v1.5 (Bioacoustics Research Program, 2017) with a “Hann” display type, the window set at Fast Fourier Transform 1,024 and viewed with a 15 s duration and 0–8 kHz frequency bandwidth, and brightness of the gray‐scale view set to 54 and contrast set to 60. To account for variation and acoustic interference from natural recording conditions, individual elements were manually selected by placing selection boxes on the spectrogram, using the waveform and visualization of the peak frequency contour to aid in refining selection boundaries. All measurements were calculated automatically by Raven for each selected element. The measurements were as follows: begin time, end time, 90% duration (duration within which 90% of the power within the signal occurs), peak frequency (frequency with the highest power), center frequency (frequency that divides the signal into two frequency intervals of equal energy), 90% bandwidth (frequency bandwidth within which 90% of the power within the signal occurs), and the peak frequency contour (a vector of the peak frequencies of each spectrogram slice in the selection; Charif et al., 2010). We used the peak frequency contour to calculate the following derived measurements: first frequency and last frequency of each element, and element slope as a function of the last frequency minus the first frequency.

We used these measurements to derive three sets of variables for analysis. Variables for each analysis were chosen to describe the physical structure of each element or song component, including the frequency, duration, and shape, while choosing measurements that are robust to natural recording conditions. All variables used within each analysis were correlated by less than 0.7.

In a preliminary analysis used to investigate the structure of the whistle song, we ran a principal component analysis (PCA) on all elements within the songs using the following variables measured from individual elements: (a) peak frequency, (b) 90% bandwidth, (c) 90% duration, and (d) element slope.

For the analysis on the song components, we used another set of variables for the introductory and final elements: (a) 90% duration, (b) peak frequency, (c) 90% bandwidth, and (d) first frequency and (e) last frequency as taken from the peak frequency contour (Table 1).

We used multi‐element‐level measurements for the analysis on the song body and full song. These were obtained by summarizing the measurements taken from the individual elements across all elements within the song or song component using the “plyr” package in R (Wickham, 2011). We calculated (a) the total duration (time from the beginning of the first element to the end of the last element, including interelement intervals), (b) highest peak frequency, (c) lowest peak frequency, (d) 90% bandwidth, (e) coefficient of variation of peak frequency (the standard deviation divided by the mean of all elements in a song; CV), (f) CV of 90% element duration, and (g) the overall slope as a function of center frequency over time (Figure 1; Table 1).

**Table 1:** Variables used for analyzing the songs and song components

Variable	Description
Element‐level variables	
90% element duration (s)	The duration of 90% of the power in the element
Peak frequency (kHz)	The frequency with the highest power in the element
90% element bandwidth (kHz)	The bandwidth that includes 90% of the power in the element
Begin frequency (kHz)	The first peak frequency in the peak frequency contour of the element
End frequency (kHz)	The last peak frequency in the peak frequency contour of the element
Song‐level variables	
Duration (s)	Beginning of the first element to the end of the last element
90% Song bandwidth (kHz)	95% frequency of the highest element in the song minus the 5% frequency of the lowest element
Highest peak frequency (kHz)	Peak frequency of the highest element in the song
Lowest peak frequency (kHz)	Peak frequency of the lowest element in the song
CV peak frequency (kHz)	The ratio of the standard deviation to the mean of peak frequencies of elements within a song
CV duration (s)	The ratio of the standard deviation to the mean of 90% element durations within a song
Song slope	A regression line of center frequency of individual elements against time

Note

All song‐level and multi‐element component variables were derived from element‐level measurements.

### 2.6 Geographic separation

We calculated a series of pairwise distances between the six study populations using ArcGIS v10.6.1 (ESRI^®^). Coordinates for each population were determined by calculating the mean of the coordinates of the focal lyrebirds in that population. Straight‐line distances were calculated in ArcGIS as the shortest straight‐line distances between coordinates.

As straight‐line distances did not always pass through known Albert's lyrebird habitat, we also calculated the length and resistance of Least Cost Paths (LCPs) in ArcGIS. To achieve this, we estimated habitat suitability using a species distribution model (SDM) and from this calculated LCPs between populations. Albert's lyrebird densities are thought to be higher in cool areas with high rainfall (Higgins et al., 2001). To capture this association with climate in the SDM, we first imported the 19 bioclimatic variables from the WorldClim database (Hijmans et al., 2005) into ArcGIS. We calculated the correlations between each climate layer and removed variables until no correlations were higher than 0.75 (as in Mason et al., 2014, Supplementary Material Appendix 1, Table S1). The remaining eight layers were “mean diurnal temperature range” (Bio2), “annual temperature range” (Bio7), “mean temperature of the warmest quarter” (Bio10), “precipitation of the wettest month” (Bio13), “precipitation seasonality” (Bio15), “precipitation of the driest quarter” (Bio17), “precipitation of the warmest quarter” (Bio18), and “precipitation of the coldest quarter” (Bio19).

Species occurrences for the SDM were obtained from the Atlas of Living Australia (ALA, 2019). Not all ALA records are expert records, and some occurrences were reported outside the known range of Albert's lyrebirds. The lyrebirds sampled in this study were found at the most northern and western extent of their range, based on expert distributions (Higgins et al., 2001), and a population at Uralba reserve in NSW (28.89°S, 153.46°E) is the most south‐eastern confirmed population (Higgins et al., 2001). We used these coordinates to determine the known extent of Albert's lyrebirds and created a buffer of 0.5° to exclude any points outside this range. This removed 40 of 5,473 records.

We used the MaxEnt prediction tool in ALA (Atlas of Living Australia, 2012) to run the SDM using the refined species occurrences and the selected eight bioclimatic variables, using 25% of records to test the model. The resulting SDM raster was imported in ArcGIS and inverted to create a measure of habitat resistance rather than suitability across the landscape (Supplementary Material Appendix 1, Figure S1). We used this “resistance” raster to create LCPs between populations using the Cost Distance Tool and Cost Path to Polyline Tool in ArcGIS. We multiplied the length of these least cost paths by the resistance measure of each to obtain weighted distance measures, which we used as measures of geographic separation between populations (as in Jensen et al., 2019).

### 2.7 Statistical analysis

#### 2.7.1 Analytical approach

**Figure 2:**
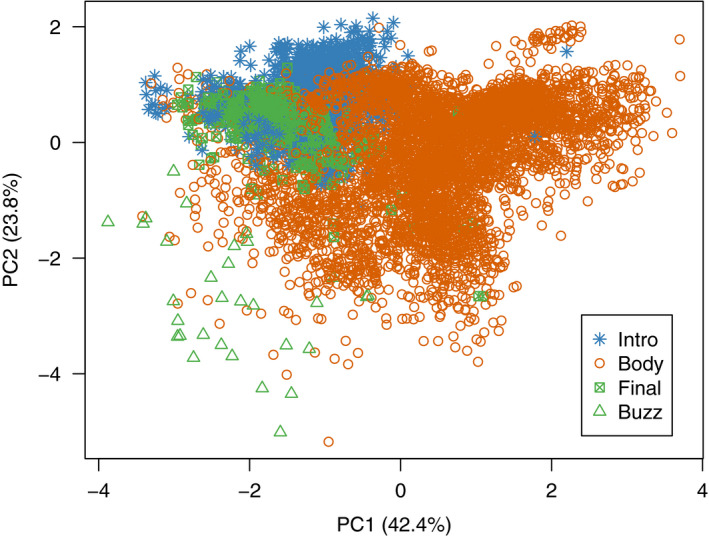
Variation among elements as defined by the first two principal components generated from a PCA on all elements (trait loadings in Supplementary Material Appendix 1, Table S2). Position within the song (intro, body, final, or buzz final elements) is represented by color and symbol. The final elements in the lower left‐hand quadrant are the “buzz” elements only sung by males of the Goomburra population (Figure 3). The body elements that overlap these buzz elements are elements of a similar acoustic quality found in the Border Ranges population. This graph includes all elements from all songs used in the full analysis (*n* = 7,269 elements)

Before conducting the full analysis, we classified song components by carrying out a PCA on all individual elements using Minitab v18.1 (Minitab, Inc 2017) to investigate how acoustic parameters might differ between elements based on position within the song. This PCA of individual elements showed that introductory elements and the final elements were clustered in acoustic space and clearly separated from other elements within the song, with the exception of a small number of final elements that had a "buzz" quality (Figure 2; Supplementary Material Appendix 1, Table S2). We therefore split the song into three components for analysis: introduction, body, and final element. We also analyzed the whistle song as a whole to look at overall patterns of geographic variation.

In the Mt Jerusalem population, seven out of 77 songs did not include the final element, instead ending on an element more characteristic of the “body” of the song. These songs were excluded from the analysis of final elements. In the Goomburra population, 32 out of 135 songs ended on a broadband “buzz” element that was qualitatively distinct from the rest of the final elements. We ran two analyses on the final elements, first including these elements (Supplementary Material Appendix 1, Text S1, Table S3), and second, in order to compare subtle differences in the final elements found in all populations, excluding these elements.

**Figure 3:**
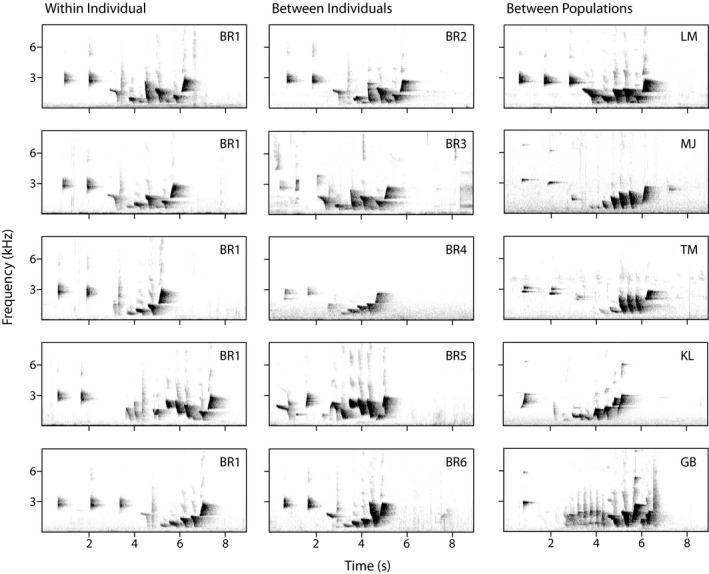
Spectrograms of example whistle songs from 11 individual males, produced with a "Hann" display and FFT 1,722. The first column (BR1) shows whistle songs from a single individual from the Border Ranges. The second column (BR2‐BR6) contains whistles from an additional five different males from the Border Ranges. The last column contains five males from an additional five different populations. BR, Border Ranges; LM, Lamington; MJ, Mt Jerusalem; TM, Tamborine Mountain; KL, Killarney; and GB, Goomburra

After classifying song components, subsequent analyses had two aims: first to determine discriminability between populations, and second to examine the relationship between acoustic and geographic distance. 90% duration of the introductory elements was positively skewed and so was transformed by taking its logarithm. End frequency of the final elements was negatively skewed and so was transformed by subtracting end frequency from 4,000 (a value greater than the highest end frequency in the dataset) and taking the logarithm of this value. All variables were centered by subtracting the variable mean from each value and scaled by dividing the centered values by the variable standard deviation, using the “scale” function in base R. During exploration of the data, whistle song variation within some individuals appeared to be noncontinuous, implying multiple whistle song variants (e.g., individual BR1 in Figure 3). Introductory and final elements were usually the same across all songs within a population, so we counted two whistle songs as different variants if the body of the songs appeared qualitatively different (to a human observer). Exploratory analysis showed that for populations with multiple clear whistle song variants, when songs were randomly selected, all apparent variations on the whistle song were captured within 30 selections. Therefore, where possible, we randomly selected 30 whistle songs from each individual to avoid excluding any within‐individual variation. For individuals with fewer than 30 whistle songs recorded (20 out of 32 individuals), we used as many high‐quality whistle songs as possible (total mean = 23.1 ± 8.40, minimum number used = 6, 77–180 songs per population, total *n* = 740). 10 songs were excluded from the analysis on full songs, eight excluded from the analysis on the song body, and an additional eight excluded from the analysis on the final elements, as the Mahalanobis distance from these songs to the centroid of all songs during PCA identified them as outliers in Minitab (Minitab, 2019). Statistical analysis was conducted on the introductory elements, the song body, the final elements, and the full song in Minitab and R v3.6.1 (R Core Team, 2017).

#### 2.7.2 Song discriminability

To determine discriminability between populations, we ran a permutated discriminant function analysis (pDFA) in R (Mundry & Sommer, 2007) using the scaled and transformed variables (Table 1). Permutated DFA is based on the function “lda” of the R package “MASS” (Venables & Ripley, 2002) but differs in accounting for unbalanced designs and repeated measures by running 1,000 DFAs on randomized data and testing for a significant difference between the discriminability of the actual data and the randomized data. This method was used to account for using multiple songs per individual. We used the pDFA to calculate the percentage of whistle songs or song components from each population that were classified into other populations (Supplementary Material Appendix 1, Table S4‐S7), and the total percentage of whistle songs or song components that were classified into the correct population.

#### 2.7.3 Acoustic and geographic distance

To determine acoustic distances between populations, we first ran a PCA on the same acoustic variables as for the pDFA (Table 1) in order to restructure the variables into a smaller set of orthogonal variables (Abdi & Williams, 2010). We then used the principal components to compute Mahalanobis distances as a measure of acoustic distance between populations using the function “pairwise.mahalanobis” from the package “HDMD” in R (McFerrin, 2013). In order to maximize the variation explained, and to standardize between models while minimizing the inclusion of less important factors, we excluded principal components that explained less than 10% of the variation (Campbell et al., 2010). Beyond this, we then kept only the number of principal components necessary to explain 75% of the total variation for the full song and the song body, and 80% of the total variation for the introductory and final elements (Supplementary material Appendix 1, Text S2). This resulted in three principal components in each model that were used to find the acoustic mean, or centroid, for each population, between which we calculated the Mahalanobis distances. Resulting acoustic distances were then compared with the straight‐line geographic distance and Least Cost Path (LCP) distances with a mantel test in the package “ade4” (Dray et al., 2007) using the Monte‐Carlo technique and 999 replicates. These acoustic distances were also used to calculate clustering of populations for visual representation as dendrograms using the “agnes” function in the package “cluster” (Maechler et al., 2019).

## 3 RESULTS

### 3.1 Qualitative results

Each population appeared to have different whistle song variants, as was evident from listening in the field and examining the spectrograms (Figure 3). Some populations had multiple whistle song variants: the Border Ranges population had at least five song variants distinguishable by eye from the spectrogram (Figure 3: columns a‐b); the Goomburra population had multiple, less distinct song variants; and the Lamington population had two clearly distinct song variants (Figure S3).

**Figure 4:**
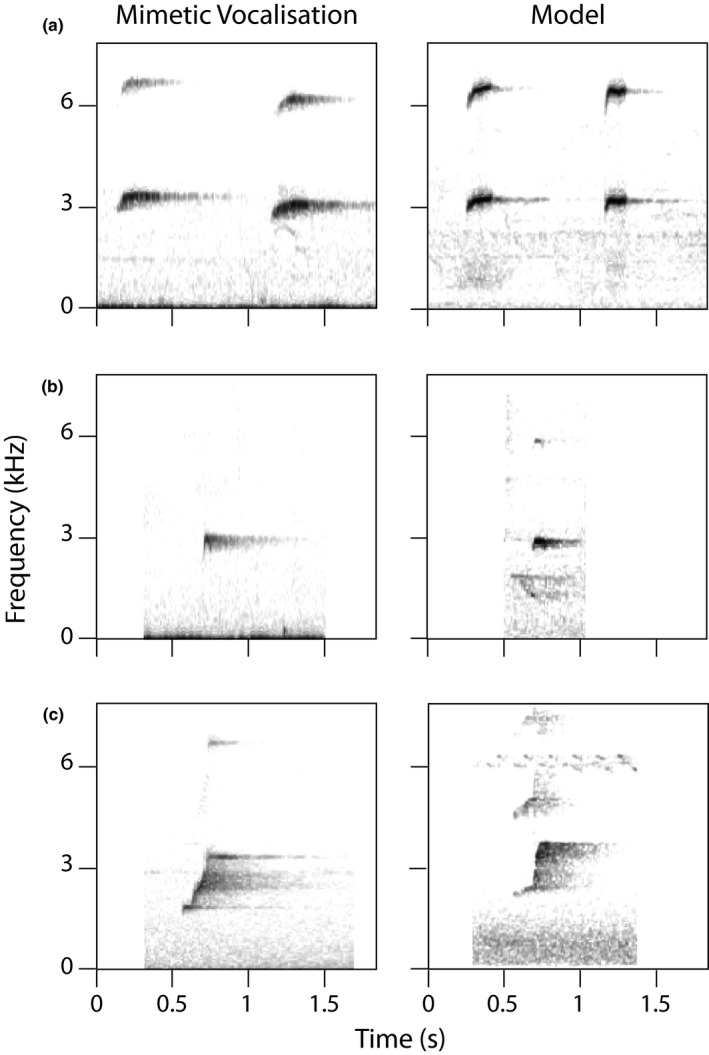
Spectrograms with examples of introductory elements and the model species likely imitated. (a) Australian king parrot (*Alisterus scapularis*), Mt Jerusalem; (b) eastern yellow robin (*Eopsaltria australis*), Goomburra; (c) grey goshawk (*Accipiter novaehollandiae*), Killarney (also used at Goomburra and, more occasionally, Border Ranges and Lamington). Recording credits for model species (a) Xeno‐canto CC XC329741 (Spencer, 2016); (b) Xeno‐canto CC XC171913 (Anderson, 2013); (c) Xeno‐canto CC SC275389 (Deoniziak, 2015)

Most whistle songs used in the analysis started with at least one introductory element. More than 95% of songs had introductory elements in the Border Ranges, Binna Burra, Mt Jerusalem, and Tamborine populations, while 87% of songs in the Goomburra population and only 54% of songs in the Killarney population had introductory elements. The acoustic structure of the introductory elements appeared to be highly consistent within populations, with the exception of the Goomburra population, which alternated between multiple types of introductory elements. In some populations, the introductory element appeared to mimic other species, including an Australian king parrot (*Alisterus scapularis*), eastern yellow robin (*Eopsaltria australis*), or grey goshawk (*Accipiter novaehollandiae*; Figure 4). In all populations, introductory elements were short, loud, broadband elements around 3 kHz (mean peak frequency 2.79 ± 0.27 kHz, mean 90% duration 0.100 ± 0.052 s, *n* = 1,120).

Most whistle songs in all populations finished with a high‐pitched ascending element (mean peak frequency 2.30 ± 0.35 kHz, *n* = 740). The population at Goomburra was the only population that varied in this: 23.7% of songs in this population finished with a broadband “buzz” element. In the other five populations, the final element was highly consistent within and between song variants (e.g., Border Ranges in Figure 3: columns a‐b).

### 3.2 Discriminability of populations

**Figure 5:**
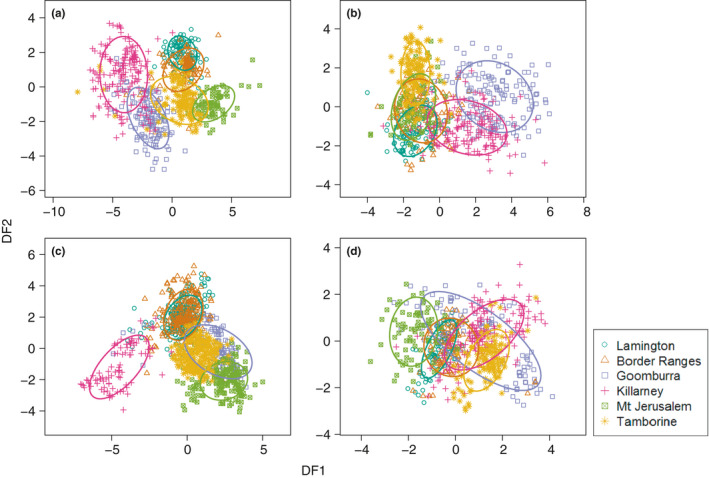
Variation in (a) full songs (*n* = 730), (b) the song body (*n* = 732), (c) introductory elements (*n* = 1,120), and (d) final elements (*n* = 693) as described by the first two discriminant functions (DF1 and DF2) from the pDFA. Ellipses are 68% confidence intervals for each population (one standard deviation from the mean). Populations are represented by symbol and color

Songs and song components were clearly distinguishable between populations (Figure 5). All individual acoustic measures of the full song differed significantly between populations (Supplementary Material Appendix 1, Table S10). A pDFA on the full songs classified 84.6% of songs into the correct populations (*p* < .001). When analyzed separately, the song body was correctly classified in 77.6% of cases (*p* < .001), the introductory elements in 76.1% of cases (*p* < .001), and the final elements in 49.5% of cases (*p* < .001).

### 3.3 Isolation by distance

Least Cost Path distances were significantly correlated with straight‐line distances (*r* ^2^ = 0.734, *p* = .013). The biggest discrepancy between the two distance measures was the distance between Tamborine and Goomburra, as the most direct route between these populations passed through highly unsuitable habitat.

**Figure 6:**
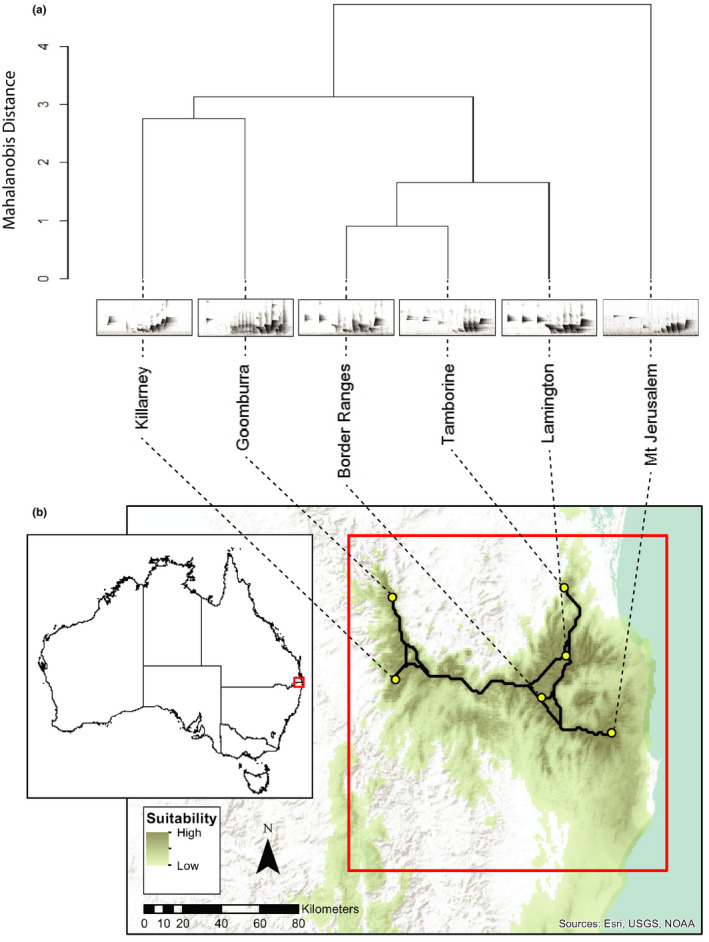
(a) Dendrogram showing the acoustic distances (Mahalanobis distances based on PCA scores from Supplementary Material Appendix 1, Table S8) among whistle songs of the sampled Albert's lyrebird populations. (b) Map showing habitat suitability from the species distribution model, with suitability values below 0.1 excluded for visualisation; lines between populations are least cost paths. The approximate extent of the Albert's lyrebird's known distribution is outlined in red

When the full song was considered, acoustic distance was significantly correlated with both straight‐line distance and LCP distance (*r* ^2^ = 0.451, *p* = .016; *r* ^2^ = 0.321, *p* = .027, respectively). This relationship was not perfect; the smallest acoustic distance was between the Border Ranges and Tamborine, which are not the most geographically proximate populations (Figure 6; Supplementary Material Appendix 1, Table S11).

The relationships between acoustic and straight‐line distance or LCP distance were also significant for the body of the song (*r* ^2^ = 0.341, *p* = .021; *r* ^2^ = 0.558, *p* = .011, respectively). Here again, the smallest acoustic distance (between Lamington and Mt Jerusalem) was not between the most geographically proximate populations.

**Figure 7:**
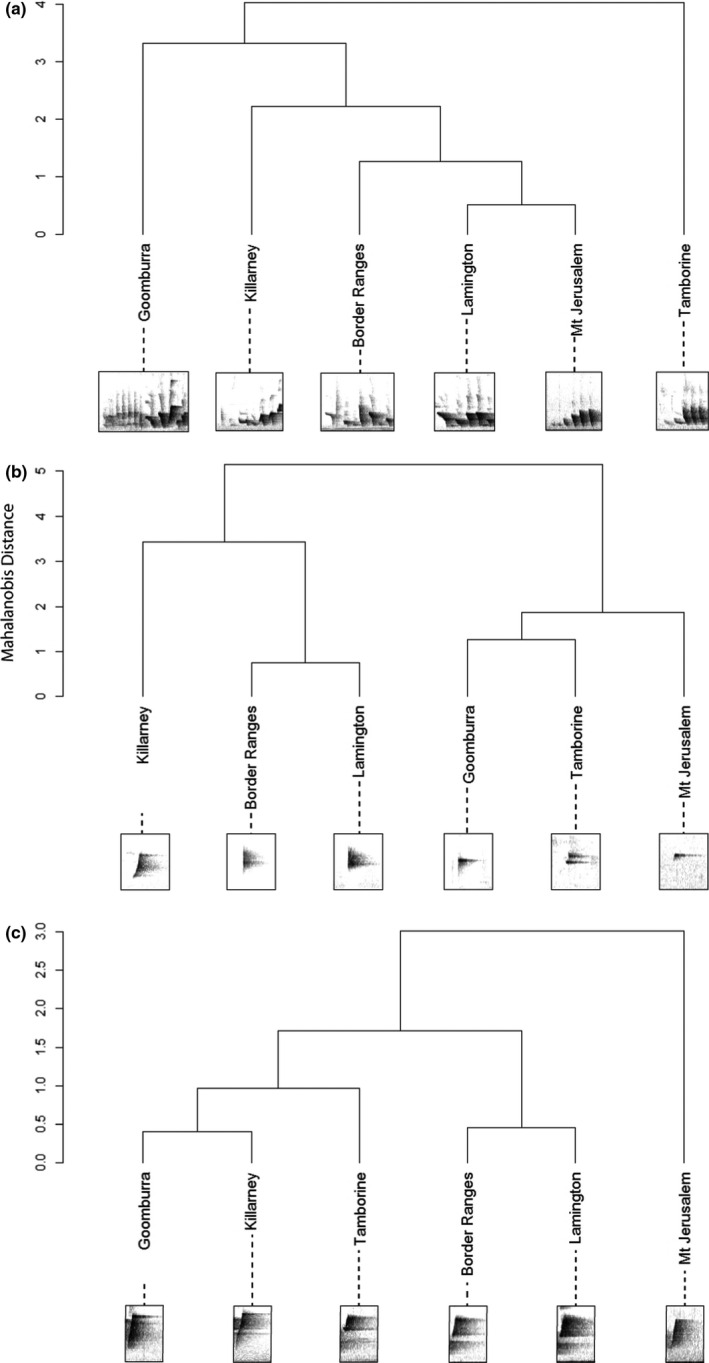
Dendograms and example spectrograms showing the acoustic distances (Mahalanobis distances based on PCA scores from Supplementary Material Appendix 1, Table S8‐S9) between populations for (a) the song body, (b) the introductory elements, and (c) the final element

Acoustic distance in the introductory elements was not correlated with either straight‐line or LCP distance (*r* ^2^ = 0.005, *p* = .458; *r* ^2^ = 0.015, *p* = .675, respectively). However, the smallest acoustic distance was between the geographically closest populations, Lamington and the Border Ranges (Figure 7b).

Acoustic distance in the final element was significantly correlated with straight‐line distance (*r* ^2^ = 0.298, *p* = .029) but not with LCP distance (*r* ^2^ = 0.037, *p* = .245; Figure 7c). After removing Tamborine mountain, acoustic distance was significantly correlated with both straight‐line distance and LCP distance (*r* ^2^ = 0.592, *p* = .019; *r* ^2^ = 0.516, *p* = .032, respectively). The same patterns were found when the “buzz” elements—unique to the Goomburra population—were included in the analysis (Supplementary Material Appendix 1, Text S1). (Figure 7a).

## 4 DISCUSSION

Albert's lyrebirds showed marked geographic variation in all components of the whistle song. Comparing songs of multiple populations revealed a three‐part structure in the song that is maintained across the entire range. Populations could be distinguished by the full song as well as the introductory elements and the body of the song, but the final element only correctly indicated the population in half of all songs. Variation in the full song was correlated with geographic separation between populations. Each component within the song showed different patterns of geographic variation, contrasting with what would be expected if they were all subject to the same processes. This demonstrates the importance of investigating spatial patterns in seemingly discrete behavioral traits at multiple levels of organization.

### 4.1 Overall geographic variation

The results of this study showed that, despite the presence of multiple whistle song variants in most populations, each population had acoustically distinct songs, quantitatively confirming the variation suggested by Robinson and Curtis (1996). The acoustic differences between populations were correlated with geographic separation, indicating that the variation in the overall song is largely affected by patterns of cultural or genetic drift (Irwin, 2000).

Acoustic distances in the full songs did not always match up with what might be expected given geographic separation between pairs of populations (Figures 6 and 7). Several explanations are possible. First, while there are positive relationships between acoustic and geographic distance overall, factors unrelated to geographic proximity such as habitat type, acoustic interference, random acoustic convergences, or localized intra‐ or intersexual selection may play a role in generating mismatches between acoustic and geographic distances (Derryberry et al., 2016; González & Ornelas, 2014; Irwin et al., 2008; Nicholls & Goldizen, 2006). Second, as cultural traits such as bird song can evolve very quickly (Mason et al., 2017), some of the signal of distance may have been lost. Third, current or past habitat corridors which could not be captured by the SDM likely influenced patterns in dispersal or social transmission. Finally, analyzing the full song likely missed some of the more detailed information on patterns and processes of variation that can be revealed by investigating components within the song (Lee et al., 2019).

### 4.2 Song components

Examining the spectrograms and the results of the PCA on all elements (Figure 3) revealed a three‐part structure in the whistle song that was present across the entire range: the introduction, the body, and the final element. A consistent overall structure, with variation in the exact elements or syllables, has been reported in other species (Barišić et al., 2018; Dalziell & Cockburn, 2008; Lee et al., 2019; Nelson & Poesel, 2007; Williams et al., 2013). This consistency of the whistle song structure, and the temporally discrete manner in which songs were produced, implies that the whistle songs are perceived as a whole unit, and makes the differing patterns of variation within the song even more interesting.

Despite the consistent structure across the range, each song component had different patterns of variation, to the point that the acoustic relationships between populations differed with song component (Figures 6 and 7). Notably, differences in the body of the song and the final element were correlated with geographic distance between populations, but the introductory element did not change with distance. This variation suggests that the different components are not affected by the same processes and are instead following separate evolutionary trajectories (Gil & Gahr, 2002; Lee et al., 2019; Nelson, 2017; Williams et al., 2013).

Introductory elements from all populations were clustered in acoustic space and largely differed from elements in the song body (Figure 2), suggesting that there are some similarities in the acoustic structure of introductory elements across populations. However, introductory elements were still distinct between populations, and most songs from a population shared the same variant of introductory element. Differences between populations were not significantly related to our geographic distance metrics. This pattern of variation between populations is perhaps not surprising if the introductory elements are, as we suspect, mimetic. Mimicry, particularly when multiple model species are involved, is unlikely to change in a continuous manner like species‐specific components of the song and would instead involve discrete changes in model species between locations. The introductory element is unlikely to be influenced by the available model species, as these species are present across the entire range. It may instead be driven by the acoustic attributes that transmit best through the local habitat type (Dalziell et al., 2015). Alternatively, the introductory element may evolve rapidly under strong cultural selection so that any geographic signal quickly becomes lost. A similar pattern of locally conserved, nonmimetic song components that vary between populations may be found in signals used to convey population membership (González & Ornelas, 2014; Nelson, 2017; Nelson & Poesel, 2007). However, introductory elements in Albert's lyrebird whistle songs are unlikely to be a dialect identifier, as individual lyrebirds may have to travel more than 30 km to encounter a different introductory element, an improbable distance for this sedentary species. Many species begin their songs with an “alerting component” that is consistent in structure and resistant to environmental degradation, alerting listeners to the more complex signal to follow (Nelson, 2017; Richards, 1981; Williams et al., 2013). However, it seems unlikely that a mimetic vocalization would perform this function. Overall, it is puzzling that whistle songs across populations appear to begin with mimetic elements that are highly consistent within populations and show similar acoustic structure across populations. Current hypotheses for mimetic vocalizations do not easily fit with these results (Dalziell et al., 2015); therefore, the development and function of mimetic introductory notes requires further investigation.

The body of the song was highly variable within individuals. Despite this, there were still considerable differences between populations, and these differences were greater with increasing geographic distance. We propose the variation in the song body is likely driven by conspecific males. Variation in the song body appeared to be noncontinuous, giving rise to discrete song variants, which were shared between neighboring males. Neighboring males often match song types in a show of aggression (Vehrencamp, 2001), or as a way to distinguish between neighbors and intruders (Beecher & Brenowitz, 2005). Such intrasexual interactions are thought to be the drivers of song repertoire size in birds with small‐medium repertoires (Catchpole & Slater, 2008). The number of whistle song variants in each lyrebird population appears to be relatively small (up to five or six, Backhouse, Pers. Obs.), particularly compared to each male's large repertoire of mimetic song types (up to 15 vocalization types from 4 to 9 model species; Robinson & Curtis, 1996). Furthermore, male lyrebirds have been observed responding to whistle songs of neighboring males with their own whistle songs, sometimes interrupting sequences of mimicry in order to do so (Robinson & Curtis, 1996, Backhouse, Pers. Obs.). These geographic patterns of variation in the song body thus support previous speculation that whistle songs are important in male–male competition (Robinson & Curtis, 1996) and suggest that explicit experimental tests of inter‐ and intrasexual functions of the whistle song are likely to be highly insightful.

The final elements of the whistle song were very similar across the species range to the extent that they could not be used to reliably distinguish among populations. Nevertheless, differences between populations in the final element were correlated with straight‐line distance and marginally correlated with the LCP distance. Removing the Tamborine population resulted in a significant correlation between acoustic distance and LCP distance. These results imply that while cultural or genetic drift may explain some variation in this element across the landscape, there are likely opposing processes keeping these elements similar across all populations (Gil & Gahr, 2002; Lee et al., 2019; Nelson, 2017). Some components of male bird song are conserved where there is directional inter‐ or intrasexual selection for acoustic characteristics at a physical performance limit that indicates male quality (Byers et al., 2010; Gil & Gahr, 2002). The final element of the lyrebird whistle song does not appear to be at a performance limit, but may contain physically challenging or preferred characteristics that we have not detected in this study.

### 4.3 Song variation and conservation

As population sizes decrease and habitat fragmentation increases, it is increasingly important to understand geographic variation in song. The song of oscine passerines, as a largely culturally transmitted trait, has a particularly high potential to change across the landscape (Parker et al., 2012). Differences between populations may increase with habitat fragmentation (Fayet et al., 2014), and so geographic variation in song can be a useful indicator of habitat connectivity, disturbance, and population viability before these can be detected through other traits such as genetic markers (Laiolo & Tella, 2005; Laiolo et al., 2008). In addition, the behavioral diversity that leads to unique cultures, such as the whistle song variants found in the Albert's lyrebird populations here, presents alternative biodiversity indicators relevant to conservation. Traditional conservation has focused on genetic and morphological diversity, but there are recent arguments that we should conserve animal “cultures” for their own sake, and what they contribute to “distinctiveness of place” (Lomolino et al., 2015). Quantifying geographic patterns of song divergence may thus help us identify “culturally significant units” across the landscape that are particularly worthy of conservation (Ryan, 2006). For example, conservation efforts on Albert's lyrebirds could focus on conserving populations containing culturally significant units such as the unique whistle songs identified in this study (e.g., Mt Jerusalem, which had the highest Mahalanobis distance from all other populations in the full song, Figure 6) to help maintain high cultural diversity across the species range. In addition, Australia's extensive 2019/2020 bushfires have affected an estimated 32% of the Albert's lyrebird habitat (Sullivan, 2020), and as such the variation in the whistle song presented here will be an invaluable framework for future investigations into the effects that these bushfires have had on the species’ cultural diversity.

### 4.4 Conclusion

This study revealed complex spatial patterns of variation among the components of a socially transmitted, species‐specific song, reinforcing the notion that single songs may contain multiple units under different evolutionary trajectories (Lee et al., 2019; Nelson, 2017; Williams et al., 2013). Knowledge of such spatial patterns of within‐song variation enables further work to determine possible selective pressures and constraints acting on each song component. For example, playback experiments using individual song components would help determine whether the components are more important for inter‐ or intrasexual communication (Leitão & Riebel, 2003), and whether they exhibit acoustic attributes that transmit best through the local habitat type (Nicholls & Goldizen, 2006). In addition, our results identify distinct vocal cultures in each of the six Albert's lyrebird populations, thus generating spatially explicit targets for cultural conservation. As such, our study highlights the importance for science and conservation management of investigating spatial patterns within seemingly discrete behavioral traits at multiple levels of organization.

## CONFLICT OF INTEREST

The authors have no conflicts of interest to declare.

## AUTHOR CONTRIBUTIONS

**Fiona Backhouse:** Conceptualization (equal); Data curation (lead); Formal analysis (lead); Funding acquisition (equal); Investigation (lead); Methodology (lead); Writing—original draft (lead); Writing—review & editing (lead). **Anastasia H. Dalziell:** Conceptualization (equal); Formal analysis (supporting); Funding acquisition (equal); Investigation (supporting); Methodology (supporting); Resources (equal); Supervision (equal); Writing—original draft (supporting); Writing—review & editing (equal). **Robert D. Magrath:** Conceptualization (equal); Formal analysis (supporting); Investigation (supporting); Methodology (supporting); Supervision (supporting); Writing—original draft (supporting); Writing—review & editing (equal). **Aaron N. Rice:** Formal analysis (supporting); Funding acquisition (equal); Methodology (supporting); Writing—review & editing (supporting). **Taylor L. Crisologo:** Data curation (supporting); Writing—review & editing (supporting). **Justin A. Welbergen:** Conceptualization (equal); Formal analysis (supporting); Funding acquisition (equal); Investigation (supporting); Methodology (supporting); Resources (equal); Supervision (equal); Writing—original draft (supporting); Writing—review & editing (equal).

## ETHICAL APPROVAL

All work for this study was approved by the Western Sydney University Animal Care and Ethics Committee (#A12077) and data collected under Scientific Research Permits from the NSW Parks and Wildlife Service (#SL101351) and the QLD Parks and Wildlife Service (#WITK18768218).

## Supporting information

Supplementary Material

## Data Availability

The data and R code used for the analysis and the species distribution model are available via the Dryad Digital Repository: https://doi.org/10.5061/dryad.w3r2280pn.
