# Supplementary material for: Differential geographic patterns in song components of male Albert’s lyrebirds
Source: Ecol Evol. 2021 Feb 14;11(6):2701–16. doi: 10.1002/ece3.7225 (PMC7981226; doi:10.1002/ece3.7225)
Supplement: Supplementary file 1 — Supplementary Material [file ECE3-11-2701-s001.docx]

**Supplementary Material: Differential geographic patterns in song components of male Albert’s lyrebirds**

Table S1. correlations between BioClim variables used in the species distribution model.

| Layer | Bio2 | Bio7 | Bio10 | Bio13 | Bio15 | Bio17 | Bio18 | Bio19 |
| --- | --- | --- | --- | --- | --- | --- | --- | --- |
| Bio2 | 1.000 | 0.015 | 0.621 | -0.104 | 0.514 | -0.372 | -0.202 | -0.242 |
| Bio7 | 0.015 | 1.000 | -0.455 | -0.589 | -0.069 | -0.375 | -0.389 | -0.475 |
| Bio10 | 0.621 | -0.455 | 1.000 | 0.303 | 0.412 | -0.054 | 0.101 | 0.112 |
| Bio13 | -0.104 | -0.589 | 0.303 | 1.000 | 0.138 | 0.429 | 0.743 | 0.584 |
| Bio15 | 0.514 | -0.069 | 0.412 | 0.138 | 1.000 | -0.515 | -0.104 | -0.274 |
| Bio17 | -0.372 | -0.375 | -0.054 | 0.429 | -0.515 | 1.000 | 0.581 | 0.699 |
| Bio18 | -0.202 | -0.389 | 0.101 | 0.743 | -0.104 | 0.581 | 1.000 | 0.374 |
| Bio19 | -0.242 | -0.475 | 0.112 | 0.584 | -0.274 | 0.699 | 0.374 | 1.000 |


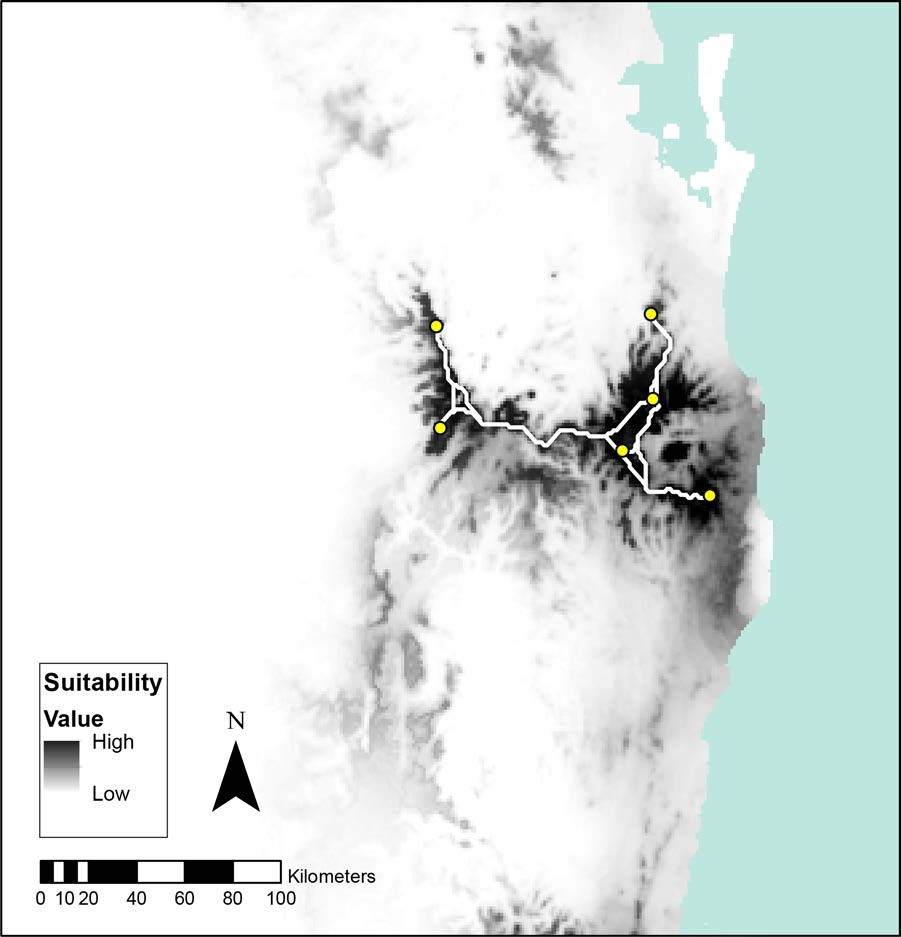


Figure S1. The species distribution raster with least cost paths between populations. The extent of this map is the extent of the area used in the SDM.

| Table S2: The factor loadings for each variable used in the PCA on all elements. | | | |
| --- | --- | --- | --- |
| **Factor loadings** | **PC1** | **PC2** | **PC3** |
| **Eigenvalue** | 1.303 | 0.976 | 0.887 |
| **Proportion explained** | 0.424 | 0.238 | 0.197 |
| **Peak frequency** | -0.613 | 0.151 | 0.096 |
| **90% bandwidth** | -0.382 | -0.742 | 0.505 |
| **Slope** | -0.489 | 0.614 | 0.292 |
| **90% duration** | 0.490 | 0.224 | 0.806 |

Text S1: Results for the final elements when including the ‘buzz’ elements.

The final elements including buzz elements were reclassified into the correct population in 50.7% of cases (p < 0.001). Acoustic distance in the final element when including buzz elements was significantly correlated with straight-line distance (r^2^ = 0.294, p = 0.036) but not with LCP distance (r^2^ = 0.061, p = 0.192).

| Table S3: The factor loadings for each variable used in the PCA on the final elements, including the ‘buzz’ elements from Goomburra. | | | |
| --- | --- | --- | --- |
| **Factor loadings** | **PC1** | **PC2** | **PC3** |
| **Eigenvalue** | 1.46 | 0.949 | 0.891 |
| **Proportion explained** | 0.423 | 0.180 | 0.159 |
| **Peak frequency** | -0.430 | -0.715 | 0.009 |
| **First frequency** | -0.543 | -0.112 | -0.087 |
| **End frequency** | 0.409 | -0.097 | -0.767 |
| **90% bandwidth** | 0.421 | -0.674 | 0.011 |
| **90% duration** | 0.418 | -0.110 | 0.636 |

Table S4. Confusion matrix from the pDFA on full songs.

|  | BB | BR | GB | KL | MJ | TM |
| --- | --- | --- | --- | --- | --- | --- |
| BB | 0.8260 | 0.2149 | 0.0009 | 0 | 0 | 0.0009 |
| BR | 0.1392 | 0.7048 | 0.0253 | 0.0114 | 0.0050 | 0.0329 |
| GB | 0.0009 | 0.0158 | 0.8740 | 0.0688 | 0.0025 | 0.0407 |
| KL | 0.0216 | 0.0160 | 0.0985 | 0.8972 | 0.0008 | 0.0478 |
| MJ | 0 | 0.0003 | 0 | 7.05E-05 | 0.8862 | 0.0085 |
| TM | 0.0123 | 0.0482 | 0.0013 | 0.0226 | 0.1055 | 0.8691 |

Table S5. Confusion matrix from the pDFA on intro elements.

|  | BB | BR | GB | KL | MJ | TM |
| --- | --- | --- | --- | --- | --- | --- |
| BB | 0.7697 | 0.2819 | 0.0161 | 0.0733 | 0 | 0.0545 |
| BR | 0.1837 | 0.6797 | 0.0073 | 0.0616 | 0 | 0.0290 |
| GB | 0.0089 | 0.0048 | 0.5940 | 0.0517 | 0.1519 | 0.0013 |
| KL | 0.0160 | 0.0036 | 0.0031 | 0.8133 | 6E-05 | 0.0010 |
| MJ | 0 | 0 | 0.1627 | 0 | 0.777 | 0.0423 |
| TM | 0.0218 | 0.0300 | 0.2167 | 0 | 0.0711 | 0.8719 |

Table S6. Confusion matrix from the pDFA on the song body.

|  | BB | BR | GB | KL | MJ | TM |
| --- | --- | --- | --- | --- | --- | --- |
| BB | 0.7612 | 0.1279 | 0.0017 | 0.0132 | 0.0558 | 0.0261 |
| BR | 0.0842 | 0.7138 | 0.0224 | 0.0145 | 0.0583 | 0.0054 |
| GB | 0.0009 | 0.0581 | 0.8490 | 0.1089 | 0.0018 | 0.0020 |
| KL | 0.1009 | 0.0348 | 0.1253 | 0.8455 | 0.1738 | 0.0457 |
| MJ | 0.0526 | 0.0654 | 0.0016 | 0.0101 | 0.4931 | 0.0759 |
| TM | 0.0003 | 0 | 0 | 0.0079 | 0.2172 | 0.8450 |

Table S7. Confusion matrix from the pDFA on the final elements.

|  | BB | BR | GB | KL | MJ | TM |
| --- | --- | --- | --- | --- | --- | --- |
| BB | 0.4591 | 0.2517 | 0.0317 | 0.0132 | 0.0885 | 0.0071 |
| BR | 0.2950 | 0.3638 | 0.1105 | 0.0352 | 0.0577 | 0.0502 |
| GB | 0.0568 | 0.0675 | 0.2596 | 0.1514 | 0.1724 | 0.1480 |
| KL | 0.0778 | 0.1864 | 0.3574 | 0.6518 | 0.0304 | 0.2394 |
| MJ | 0.0948 | 0.0348 | 0.0059 | 0.0042 | 0.6500 | 0.0036 |
| TM | 0.0166 | 0.0958 | 0.2348 | 0.1442 | 0.0009 | 0.5518 |

Text S2. Principal Component Analysis

The first three principal components were kept for the full song and for each song component. For the full songs, these explained 78.4% of the total variation. Most populations could be clearly separated by PC1 and PC2 (Fig. S2a). The first three principal components in the analysis on the song body explained 79.3% of the total variation. Goomburra and Tamborine could be more easily distinguished by PC1 and PC2 than the other populations (Fig. S2b).

The three principal components retained in the analysis on introductory elements explained 89.5% of the total variation. Most populations could be clearly distinguished by the first two principal components (Fig. S2c). The first three principal components in the analysis on the final elements explained 84.4% of the total variation. Goomburra showed considerable variation across PC1; most populations were not easily distinguished by PC1 and PC2 (Fig. SA2d). Over half of the variance in these two models was captured in the first principal component.


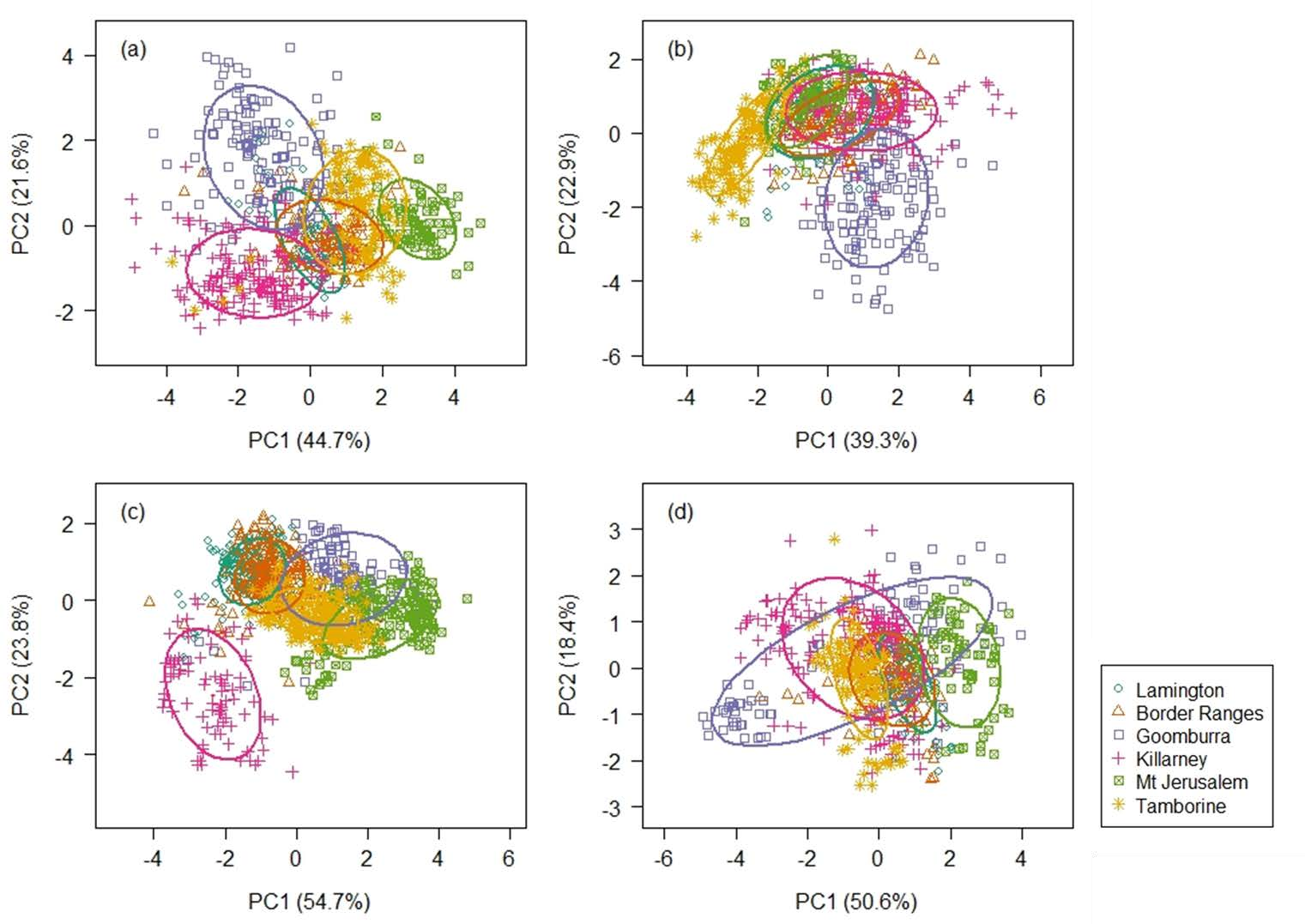


Figure S2. Variation in (a) full songs (n = 730), (b) the song body (n = 732), (c) introductory elements (n = 1120) and (d) final elements (n = 693) as described by the first two principal components. Ellipses are 68% confidence intervals for each population (one standard deviation from the mean). Populations are represented by symbol and colour.

Table S8. The factor loadings for each variable in the PCA on the full song and the PCA on the song body. The same variables were used for both analyses.

|  | **Full Song** |  |  | **Song Body** |  |  |
| --- | --- | --- | --- | --- | --- | --- |
| **Factor loadings** | **PC1** | **PC2** | **PC3** | **PC1** | **PC2** | **PC3** |
| **Eigenvalue** | 3.13 | 1.51 | 0.953 | 2.75 | 1.61 | 1.20 |
| **Proportion explained** | 0.447 | 0.216 | 0.122 | 0.393 | 0.229 | 0.171 |
| **Song duration** | 0.373 | 0.278 | -0.556 | 0.486 | -0.142 | 0.119 |
| **Song bandwidth** | 0.466 | -0.184 | -0.104 | 0.515 | 0.023 | -0.120 |
| **Max**  **frequency** | 0.366 | 0.356 | 0.084 | 0.521 | -0.125 | -0.008 |
| **Min frequency** | -0.215 | 0.715 | -0.022 | 0.045 | -0.741 | -0.075 |
| **CV peak frequency** | 0.453 | -0.360 | 0.154 | 0.274 | 0.641 | -0.023 |
| **CV**  **duration** | -0.278 | -0.270 | -0.789 | 0.285 | -0.052 | 0.698 |
| **Slope** | -0.425 | -0.220 | 0.162 | -0.262 | 0.020 | 0.691 |

Table S9. The factor loadings for each variable in the PCA on the introductory elements and the PCA on the final elements. The same variables were used for both analyses.

|  | **Introductory elements** | |  | **Final elements** | |  |
| --- | --- | --- | --- | --- | --- | --- |
| **Factor loadings** | **PC1** | **PC2** | **PC3** | **PC1** | **PC2** | **PC3** |
| **Eigenvalue** | 2.73 | 1.19 | 0.553 | 2.53 | 0.920 | 0.773 |
| **Proportion explained** | 0.547 | 0.238 | 0.111 | 0.506 | 0.184 | 0.155 |
| **Peak frequency** | 0.545 | -0.087 | -0.233 | -0.401 | 0.290 | -0.236 |
| **First frequency** | 0.459 | 0.468 | 0.016 | -0.574 | -0.194 | -0.219 |
| **End frequency** | 0.450 | -0.402 | -0.573 | 0.301 | -0.835 | -0.022 |
| **90%**  **bandwidth** | -0.442 | -0.465 | -0.376 | 0.538 | 0.416 | 0.218 |
| **90%**  **duration** | 0.306 | -0.628 | 0.689 | 0.360 | 0.091 | -0.921 |


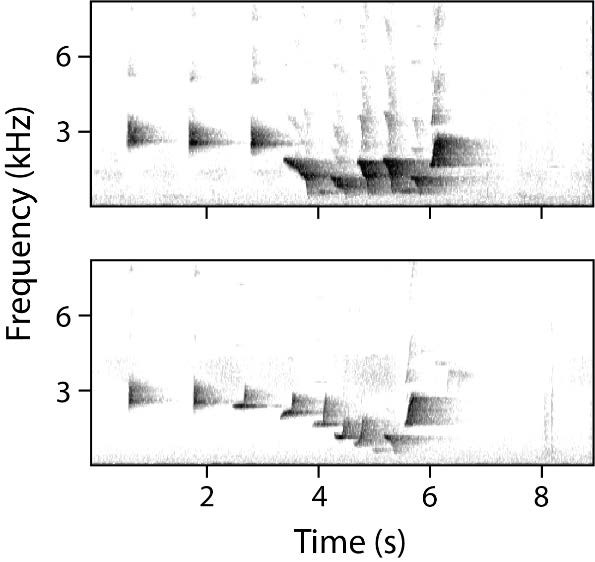


Figure S3. Examples of the two song types found at Lamington (Binna Burra). Both types have identical introductory and final elements, but vary in the body of the song.

Table S10. Summary statistics for the full songs from each location. Values are mean (± standard deviation). Differences between populations were tested with a one-way ANOVA.

| Location | Lamington | Border Ranges | Goomburra | Killarney | Mt  Jerusalem | Tamborine | ANOVA  result  (df=5,724) |
| --- | --- | --- | --- | --- | --- | --- | --- |
| Duration  (s) | 5.169  (±0.671) | 4.879  (±1.041) | 4.874  (±0.941) | 3.865  (±0.957) | 5.354  (±0.687) | 5.844  (±0.964) | F=79.60 P<0.001 |
| Song bandwidth (kHz) | 2.448  (±0.125) | 2.588  (±0.161) | 2.373  (±0.233) | 2.461  (±0.218) | 2.861  (±0.099) | 2.638  (±0.142) | F=94.21 P<0.001 |
| Max  frequency (kHz) | 2.644  (±0.065) | 2.766  (±0.121) | 2.959  (±0.193) | 2.616  (±0.312) | 3.346  (±0.075) | 2.923  (±0.183) | F=180.33 P<0.001 |
| Min frequency (kHz) | 0.714  (±0.138) | 0.732  (±0.089) | 1.065  (±0.218) | 0.699  (±0.116) | 0.684  (±0.091) | 0.798  (±0.133) | F=134.81 P<0.001 |
| CV peak frequency (kHz) | 0.412  (±0.047) | 0.434  (±0.047) | 0.305  (±0.05) | 0.379  (±0.053) | 0.513  (±0.057) | 0.461  (±0.048) | F=227.23 P<0.001 |
| CV  duration (s) | 0.605  (±0.177) | 0.475  (±0.119) | 0.557  (±0.188) | 0.639  (±0.148) | 0.348  (±0.075) | 0.369  (±0.114) | F=83.70 P<0.001 |
| Slope | -11.73  (±2.643) | -9.786  (±4.500) | -0.928  (±3.677) | 7.010  (±5.139) | -16.41  (±3.894) | -6.283  (±6.176) | F=418.79 P<0.001 |

Table S11. Distances between all pairs of populations. The length of least cost paths weighted by resistance and the straight line distances were compared with the acoustic distances in mantel tests to determine the relationship between acoustic and geographic distance.

| Locations | | Geographic measures | | | | Mahalanobis distances from PCA scores | | |  |
| --- | --- | --- | --- | --- | --- | --- | --- | --- | --- |
| Site X | Site Y | Resistance of least cost path | Length of least cost path | Length of least cost path weighted by resistance | Straight line distance | Acoustic distance of full songs | Acoustic distance of intro elements | Acoustic distance of song body | Acoustic distance of final elements |
| Lamington | Border Ranges | 0.014 | 31.682 | 0.434 | 25.009 | 0.930 | 0.747 | 0.814 | 0.466 |
| Lamington | Goomburra | 0.183 | 135.032 | 24.698 | 95.067 | 2.363 | 2.559 | 2.877 | 1.613 |
| Lamington | Killarney | 0.153 | 110.887 | 16.967 | 89.181 | 1.886 | 3.420 | 1.924 | 1.719 |
| Lamington | Mt Jerusalem | 0.040 | 74.145 | 2.966 | 46.519 | 3.222 | 3.808 | 0.5178 | 1.547 |
| Lamington | Tamborine | 0.073 | 40.799 | 2.984 | 35.372 | 1.644 | 2.093 | 2.429 | 1.410 |
| Border Ranges | Goomburra | 0.177 | 117.679 | 20.841 | 93.361 | 2.636 | 2.130 | 2.683 | 1.192 |
| Border Ranges | Killarney | 0.147 | 93.535 | 13.769 | 76.360 | 2.257 | 3.422 | 2.226 | 1.289 |
| Border Ranges | Mt Jerusalem | 0.0332 | 50.274 | 1.670 | 40.719 | 2.493 | 3.359 | 1.270 | 1.915 |
| Border Ranges | Tamborine | 0.086 | 71.640 | 6.172 | 58.178 | 0.910 | 1.770 | 3.092 | 1.073 |
| Goomburra | Killarney | 0.054 | 46.287 | 2.506 | 42.676 | 2.746 | 4.763 | 2.560 | 0.406 |
| Goomburra | Mt Jerusalem | 0.210 | 167.260 | 35.128 | 133.863 | 4.560 | 1.489 | 3.323 | 3.005 |
| Goomburra | Tamborine | 0.256 | 174.446 | 44.601 | 89.352 | 2.868 | 1.271 | 4.015 | 0.581 |
| Killarney | Mt  Jerusalem | 0.180 | 143.115 | 25.778 | 115.513 | 4.706 | 5.142 | 2.120 | 2.977 |
| Killarney | Tamborine | 0.226 | 150.301 | 33.935 | 99.589 | 3.130 | 3.602 | 3.462 | 0.969 |
| Mt Jerusalem | Tamborine | 0.112 | 114.103 | 12.831 | 79.266 | 1.766 | 1.864 | 2.168 | 2.914 |
